# Liraglutide and Βeta‐Cell Function in Young Adults With Long‐Standing Type 1 Diabetes and Residual C‐Peptide: A Blinded, Randomized Controlled Trial

**DOI:** 10.1111/dom.71204

**Published:** 2026-08-13

**Authors:** José Caballero‐Corbalán, Per Lundkvist, Daniel Espes, Per‐Ola Carlsson

**Affiliations:** ^1^ Department of Medical Sciences Uppsala University Uppsala Sweden; ^2^ Department of Medical Cell Biology Uppsala University Uppsala Sweden; ^3^ Science for Life Laboratory Uppsala University Uppsala Sweden

**Keywords:** beta‐cell function, C‐peptide, GLP‐1 receptor agonist, liraglutide, type 1 diabetes

## Abstract

**Aims:**

To evaluate the effect of liraglutide on residual beta‐cell function in adults with long‐standing type 1 diabetes (T1D) and detectable C‐peptide.

**Materials and Methods:**

In this randomized, blinded, placebo‐controlled trial, 18 adults with T1D duration ≥ 5 years and detectable fasting C‐peptide were assigned to liraglutide or placebo for 52 weeks. Mixed‐meal tolerance tests were performed at baseline and weeks 6, 52, and 65. The primary endpoint was change in stimulated C‐peptide area under the curve from baseline to week 52.

**Results:**

Liraglutide did not improve stimulated C‐peptide compared with placebo at week 52. In exploratory post hoc longitudinal analyses including the post‐treatment period (weeks 0–65), stimulated C‐peptide declined more steeply in the liraglutide group than in the placebo group, accompanied by increasing insulin requirements over time.

**Conclusions:**

In adults with long‐standing T1D, liraglutide did not preserve stimulated C‐peptide after 52 weeks. Exploratory post hoc longitudinal analyses suggested a steeper decline in stimulated C‐peptide with liraglutide than with placebo.

## 1 Introduction

Residual C‐peptide secretion in long‐standing T1D is prevalent and associated with improved metabolic stability, lower hypoglycemia risk and reduced complications [1, 2, 3], underscoring its clinical relevance beyond the early disease phase.

Glucagon‐like peptide‐1 receptor agonists (GLP‐1RAs) can reduce body weight, insulin requirements and improve glycemic control in T1D and are increasingly used off‐label [4, 5, 6, 7]. In recent‐onset T1D, liraglutide has shown modest short‐term preservation of stimulated C‐peptide during active treatment, with loss of effect after withdrawal [8].

However, randomized data on GLP‐1RA therapy and beta‐cell function in long‐standing T1D are limited, and prior trials have rarely incorporated post‐treatment assessments [9]. The effects of adjunctive GLP‐1RA therapy in established T1D therefore remain uncertain.

We conducted a randomized, placebo‐controlled trial to evaluate the effect of adjunctive liraglutide on MMTT‐stimulated C‐peptide area under the curve (AUC) in adults with long‐standing T1D.

## 2 Materials and Methods

### 2.1 Study Design and Participants

This investigator‐initiated, randomized, placebo‐controlled, parallel‐group, single‐center clinical trial in Uppsala, Sweden, enrolled adults ≥ 18 years with T1D duration ≥ 5 years, HbA1c 45–75 mmol/mol, and detectable fasting C‐peptide > 1.5 pmol/L. Participants were randomized 1:1 to liraglutide or matching placebo. The randomization sequence was computer‐generated using fixed block sizes by an independent contract research organization (Clinical Trial Consultants AB, Uppsala, Sweden) and concealed using sequentially numbered, sealed envelopes. Study physicians enrolled participants and assigned them to the next available study medication according to the concealed allocation sequence. Participants, investigators, outcome assessors, and laboratory personnel remained blinded to treatment allocation throughout the study. The trial was terminated prematurely because recruitment did not reach the prespecified sample size.

The study was approved by the Swedish Ethical Review Authority (dnr 2015/341), conducted in accordance with the Declaration of Helsinki and ICH‐GCP guidelines, and registered on ClinicalTrials.gov (NCT02617654). All participants provided written informed consent. The CONSORT 2010 checklist is provided in Table S4.

### 2.2 Intervention and Study Procedures

Liraglutide was initiated at 0.6 mg daily and titrated weekly to 1.8 mg/day as tolerated; placebo followed an identical schedule. Participants continued basal–bolus insulin regimens without protocol‐mandated dosing algorithms.

Standardized MMTTs were performed at baseline and weeks 6, 52, and 65, the latter representing a prespecified post‐treatment visit 13 weeks after study drug discontinuation, as previously described [10]. C‐peptide was measured using a validated ultrasensitive ELISA (Mercodia AB, Uppsala, Sweden).

### 2.3 Outcomes

The primary endpoint was change in MMTT‐stimulated C‐peptide AUC from baseline to week 52.

Prespecified secondary endpoints included HbA1c, total daily insulin dose (U/kg/day), fasting plasma glucose, episodes of patient‐documented level 2 hypoglycemia (blood glucose < 3.0 mmol/L), and adverse events. Exploratory post hoc analyses included longitudinal analyses of MMTT‐stimulated C‐peptide across all study visits, as well as analyses of changes in body weight and BMI.

Although glucose variability was a prespecified secondary outcome, analyses were not performed because continuous glucose monitoring data were incomplete for a substantial proportion of participants.

### 2.4 Statistical Analysis

Between‐group differences at week 52 (primary endpoint) were assessed using ANCOVA adjusting for baseline values. C‐peptide AUC was analysed on the original scale without transformation. Longitudinal outcomes were analysed using linear mixed‐effects models including week (continuous), treatment group, and their interaction as fixed effects and participant‐specific random intercepts. Patient‐documented episodes of level 2 hypoglycemia were analysed using Poisson regression.

All participants with at least one post‐baseline measurement were included (modified intention‐to‐treat). No imputation was performed. The primary endpoint was analysed as prespecified. Secondary analyses should be interpreted cautiously. Exploratory post hoc longitudinal analyses should be regarded as hypothesis‐generating. *p*‐values are nominal and not adjusted for multiplicity.

### 2.5 Sample‐Size Calculation

Assuming a common standard deviation of change in MMTT‐stimulated C‐peptide AUC of 7.5%–9.7%, based on repeated MMTTs in a previous study [11] and baseline–follow‐up correlations of 0.7–0.5, a sample size of 25 participants per group was estimated to provide 80% power (two‐sided α = 0.05) to detect a between‐group difference in change in stimulated C‐peptide AUC of 6.1%–7.8%. Calculations were performed using G*Power version 3.1. Because recruitment did not reach the planned sample size, the study was underpowered to detect small or moderate between‐group differences.

## 3 Results

### 3.1 Participants

Eighty‐eight individuals were screened (Figure 1); 18 were randomized to liraglutide (*n* = 10) or placebo (*n* = 8). Sixteen participants completed the 52‐week treatment period and the 13‐week post‐treatment follow‐up and were included in the primary endpoint analysis. Two participants discontinued liraglutide during dose titration due to gastrointestinal intolerance. Baseline characteristics were generally comparable between groups (Table 1). Recruitment took place between January 2016 and March 2019. Study follow‐up was completed in September 2020. Recruitment challenges led to premature trial termination before the planned sample size was reached.

**FIGURE 1:**
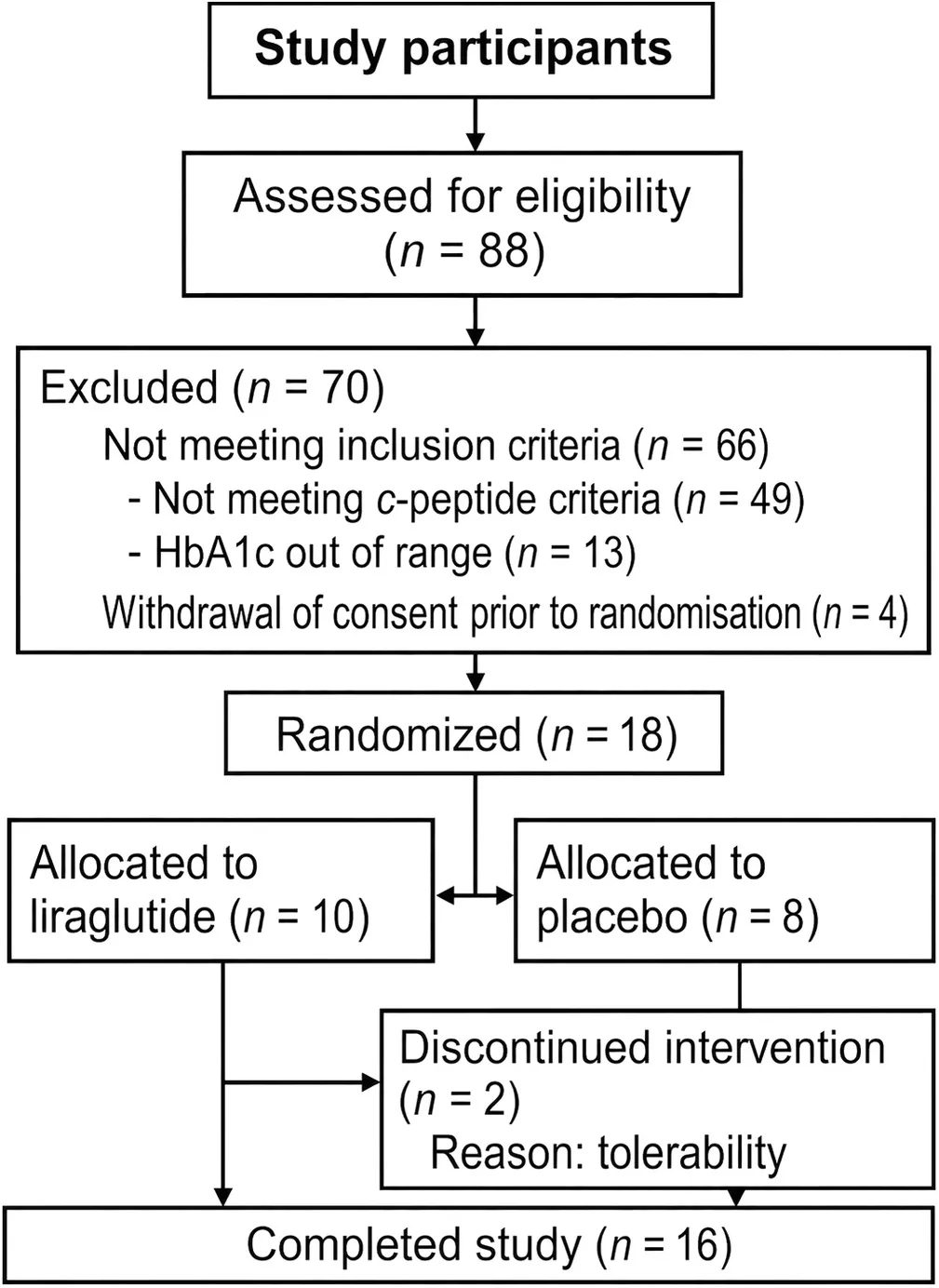
Flow of participants through the randomized, blinded, placebo‐controlled trial. Of 88 individuals assessed for eligibility, 70 were excluded, primarily due to not meeting inclusion criteria (*n* = 66), including insufficient C‐peptide at screening (*n* = 49) and HbA1c outside the permitted range (*n* = 13). Four participants withdrew consent prior to randomization. Eighteen participants were randomized to liraglutide (*n* = 10) or placebo (*n* = 8). Two participants in the liraglutide group discontinued the intervention during titration because of gastrointestinal intolerance. Sixteen participants completed the 52‐week treatment period and the 65‐week follow‐up.

**TABLE 1:** Baseline characteristics of study participants.

Variable	Placebo (*n* = 8)	Liraglutide (*n* = 10)
Male sex (%)	62	80
Age at baseline (y)	24.6 (3.4)	24.0 (3.5)
Age at diabetes onset (y)	15.6 (4.7)	14.0 (2.4)
T1D duration (y)	9.0 (3.5)	10.0 (4.8)
Weight (kg)	76.7 (15.3)	75.9 (7.5)
BMI (kg/m^2^)	25.7 (3.7)	24.1 (2.6)
Pump treatment (%)	50	10
Retinopathy (%)	25	20
HbA1c (mmol/mol)	59.9 (8.1)	54.3 (7.4)
Fasting C‐peptide (pmol/L)	25.3 (45.8)	40.1 (37.6)
Fasting glucose (mmol/L)	8.1 (2.6)	8.8 (3.1)

*Note:* Values are mean (SD) or percentages as indicated.

### 3.2 C‐Peptide

At week 52, liraglutide did not improve MMTT‐stimulated C‐peptide AUC compared with placebo (adjusted difference −537 pmol·L^−1^·min; 95% CI −5415 to 4342; *p* = 0.82) (Table 2).

**TABLE 2:** Model‐based treatment effects for primary, secondary, and exploratory post hoc outcomes.

Outcome	Adjusted estimate (95% CI)	*p*
C‐peptide AUC (pmol·L^−1^·min)^a^	−537 (−5415 to 4342)	0.820
HbA1c (mmol/mol)^a^	−7.5 (−14.5 to 0.5)	0.053
Body weight (kg)^a^	−5.9 (−9.8 to −2.0)	0.007
BMI (kg/m^2^)^a^	−2.05 (−3.17 to −0.93)	0.002
Insulin dose (U/kg/day per week)^b^	+0.0025 (0.0001 to 0.0049)	0.044

*Note:* All estimates are liraglutide minus placebo. *p*‐values are nominal and not adjusted for multiplicity.

^a^ Between‐group differences at Week 52 derived from ANCOVA models adjusted for baseline values.

^b^ Estimate represents the week × group interaction term from a linear mixed‐effects model (week modelled as continuous; participant as a random effect).

At baseline, peak stimulated C‐peptide ≥ 200 pmol/L during the MMTT was present in 4/8 (50%) participants in the liraglutide group and 2/8 (25%) in the placebo group.

In exploratory post hoc longitudinal analyses over weeks 0–65, C‐peptide declined more steeply in the liraglutide group (slope difference −85 pmol·L^−1^·min per week; 95% CI −155 to −16; *p* = 0.020).

However, on‐treatment slope‐differences (weeks 0–52 and 6–52) did not differ between groups (*p* = 0.29 and *p* = 0.08, Figure 2A).

**FIGURE 2:**
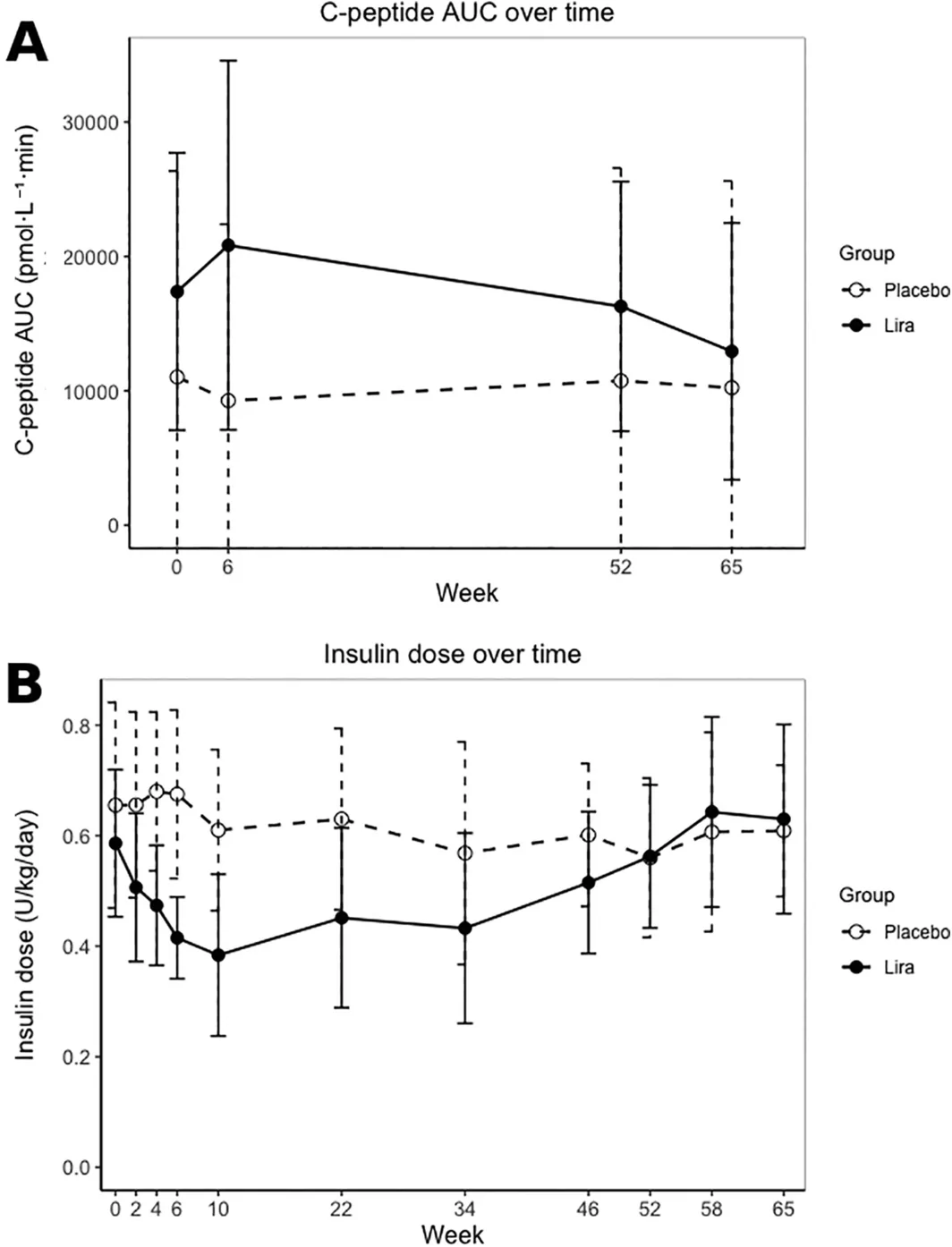
C‐peptide secretion and insulin dose over time. (A) C‐peptide area under the curve (AUC) during mixed‐meal tolerance tests (MMTT) at weeks 0, 6, 52, and 65 in adults with long‐standing type 1 diabetes randomized to liraglutide (solid line, closed circles) or placebo (dashed line, open circles). (B) Total daily insulin dose (U/kg/day) from baseline to week 65. Values are mean±95% CI based on all available observations at each visit. Week 65 represents the off‐treatment visit 13 weeks after study drug discontinuation. Analyses were performed as described in Methods (Table 2). All *p*‐values are nominal and not adjusted for multiplicity.

### 3.3 Insulin Requirements

Total daily insulin dose was lower in the liraglutide group during active treatment and gradually increased over time, resulting in a significant week × group interaction over the full study period (+0.0025 U/kg/day per week; 95% CI 0.0001 to 0.0049; *p* = 0.044) (Figure 2B).

### 3.4 HbA1c And Body Weight

At week 52, the adjusted between‐group difference in change from baseline in HbA1c was −7.5 mmol/mol (≈0.7%; 95% CI −14.5 to 0.5; *p* = 0.053). HbA1c trajectories did not differ significantly between groups over weeks 0–52 (no significant week × group interaction; Figure S1).

Participants receiving liraglutide experienced greater weight loss than those receiving placebo. At week 52, the adjusted between‐group difference in change from baseline in body weight was −5.9 kg (95% CI −9.8 to −2.0; *p* = 0.0066; Table 2; Figure S2).

Additional fasting glucose results and descriptive unmodelled values at baseline and week 52 are presented in Tables S1 and S2, respectively.

### 3.5 Safety

Rates of level 2 hypoglycemia did not differ between groups. Gastrointestinal adverse events were numerically more frequent in the liraglutide group and led to two treatment discontinuations. No severe hypoglycemia, diabetic ketoacidosis, or pancreatitis occurred. Additional safety data are presented in Table S3.

## 4 Discussion

In this randomized trial in adults with long‐standing T1D, liraglutide did not improve stimulated C‐peptide at week 52. Exploratory post hoc longitudinal analyses showed a steeper decline in C‐peptide over weeks 0–65, paralleled by increasing insulin requirements. The slope difference over weeks 0–65 included the post‐treatment phase and was directionally similar across alternative time windows. These exploratory findings should be interpreted cautiously.

The mechanisms underlying the observed longitudinal pattern remain uncertain. Although the overall difference reached statistical significance only when the post‐treatment period was included, the direction of effect was consistent across alternative time windows. The present study was not designed to determine whether the post‐treatment divergence reflects loss of a treatment‐associated effect or altered residual beta‐cell function.

Our findings are consistent with earlier liraglutide studies in long‐standing T1D, such as ADJUNCT ONE and ADJUNCT TWO, which reported no improvement in stimulated C‐peptide during treatment [4, 5]. They also extend recent data from NewLira and the IL‐21/liraglutide‐trial showing short‐term preservation of stimulated C‐peptide that diminishes once therapy is withdrawn [8, 12]. By incorporating repeated MMTTs at four timepoints, our study provides longitudinal assessment of stimulated C‐peptide before, during, and after treatment withdrawal. Unlike previous liraglutide studies that compared stimulated C‐peptide only with pretreatment baseline, our study also evaluated trajectories from the time participants had reached the target dose (week 6), allowing assessment of steady‐state treatment effects.

Study limitations merit consideration. The sample size was smaller than originally planned, reducing power to detect small or moderate treatment effects. Although residual beta‐cell function was limited, baseline stimulated C‐peptide was numerically higher in the liraglutide group, and regression to the mean may therefore have contributed to the observed longitudinal pattern despite adjustment for baseline values. The observed divergence in C‐peptide trajectories should be evaluated in larger, adequately powered trials.

In adults with long‐standing T1D, liraglutide did not preserve stimulated C‐peptide after 52 weeks. Exploratory post hoc longitudinal analyses showed a steeper decline in C‐peptide in the liraglutide group, accompanied by increasing exogenous insulin requirements. These results do not support a beneficial effect of liraglutide on residual beta‐cell function in established type 1 diabetes and are relevant given the increasing off‐label use of GLP‐1 receptor agonists in this population.

## Author Contributions

J.C.‐C., D.E. and P.‐O.C. wrote the study protocol. J.C.‐C., D.E., P.‐O.C. and P.L. served as study physicians and contributed to data acquisition and clinical oversight. P.L. performed the statistical analyses and created the figures and tables. J.C.‐C., D.E., P.‐O.C. and P.L. jointly interpreted the data and wrote and critically revised the manuscript. All authors approved the final version.

## Funding

This work was supported by Regionala Forskningsrådet Uppsala/Örebro, Svenska Sällskapet för Medicinsk Forskning and Göran Gustafssons Stiftelse för Naturvetenskaplig och Medicinsk Forskning.

## Conflicts of Interest

Daniel Espes is a minority shareholder in Novo Nordisk. The marketing authorisation holder Novo Nordisk provided the investigational drug (liraglutide and placebo) and financial support for trial registration and monitoring but was not involved in study design, data collection, data interpretation, data analysis or writing of the report. The other authors declare no conflicts of interest.

## Supporting information


**Table S1:** Secondary outcomes not included in the main efficacy table.
**Table S2:** Descriptive outcomes (mean ± SD).
**Table S3:** Adverse events by system category and treatment group.
**Figure S1:** HbA1c over time. HbA1c (mmol/mol) from baseline to week 65 in participants randomized to liraglutide (solid line, closed circles) or placebo (dashed line, open circles). Values represent mean±95% CI based on all available measurements at each visit (weeks 0, 2, 4, 6, 10, 22, 34, 46, 52, 58 and 65). Liraglutide was associated with lower mean HbA1c across the study period, whereas trajectories were broadly parallel between groups. Inferential analyses were performed using a linear mixed‐effects model with random intercepts and a week × group interaction term (Table 2); *p*‐values are exploratory and not adjusted for multiplicity.
**Figure S2:** Body weight over time. Body weight (kg) at weeks 0, 6, 52 and 65 in participants receiving liraglutide (solid line, closed circles) or placebo (dashed line, open circles). Values are mean±95% CI for all available observations at each time point. Liraglutide was associated with weight loss during the treatment period with partial regain after discontinuation, whereas weight remained relatively stable with placebo. The adjusted between‐group difference in change from baseline to week 52 was −5.9 kg (95% CI −9.8 to −2.0; *p* = 0.0066) in an ANCOVA model (Table 2); *p*‐values are exploratory and not adjusted for multiplicity.

**Data S1:** CONSORT 2010 checklist of information to include when reporting a randomized trial.

## Data Availability

The datasets generated and analysed during the current study are not publicly available but are available from the corresponding author on reasonable request, subject to appropriate data‐sharing agreements.
