# Supplementary material for: Liraglutide and Βeta‐Cell Function in Young Adults With Long‐Standing Type 1 Diabetes and Residual C‐Peptide: A Blinded, Randomized Controlled Trial
Source: Diabetes Obes Metab. 2026 Aug 13;28(11):10093–8. doi: 10.1111/dom.71204 (PMC13646360; doi:10.1111/dom.71204)
Supplement: Supplementary file 1 [file DOM-28-10093-s001.docx]

**SUPPLEMENT**

**Supplementary Table S1. Secondary outcomes not included in the main efficacy table**

| **Outcome** | **Adjusted estimate (95% CI)** | **P value** |
| --- | --- | --- |
| Fasting glucose¹ (mmol/L) | 1.28 (−0.97, 3.54) | 0.24 |

# Supplementary Table S2. Descriptive outcomes (mean ± SD)

Descriptive (unmodelled) values at baseline (week 0) and week 52 for key exploratory outcomes.

| **Outcome** | **Placebo baseline** | **Liraglutide baseline** | **Placebo week 52** | **Liraglutide week 52** |
| --- | --- | --- | --- | --- |
| C-peptide AUC (pmol·L⁻¹·min) | 11029 ± 18336 | 17388 ± 12337 | 10746 ± 18937 | 16285 ± 11107 |
| HbA1c (mmol/mol) | 59.9 ± 8.1 | 53.0 ± 7.8 | 60.4 ± 6.1 | 53.3 ± 8.1 |
| Body weight (kg) | 76.7 ± 15.3 | 74.9 ± 7.3 | 78.2 ± 14.5 | 70.7 ± 6.5 |
| BMI (kg/m²) | 25.2 ± 4.2 | 24.4 ± 2.8 | 25.7 ± 3.6 | 23.0 ± 2.0 |
| Insulin dose (U/kg/day) | 0.66 ± 0.22 | 0.59 ± 0.16 | 0.60 ± 0.16 | 0.56 ± 0.15 |
| Fasting glucose (mmol/L) | 8.14 ± 2.61 | 8.54 ± 3.36 | 8.45 ± 2.32 | 9.74 ± 1.63 |

*Adjusted estimates reflect between-group differences (liraglutide minus placebo) in change from baseline to week 52.
¹ ANCOVA model.
² Linear mixed-effects model (week×group interaction).
³ Descriptive means; inferential statistics for insulin dose are presented in Table 2 (LMM slope).*

**Supplementary Table S3. Adverse events by system category and treatment group**

| **System category** | **Representative events** | **Liraglutide subjects n (%)** | **Liraglutide events (n)** | **Placebo subjects n (%)** | **Placebo events (n)** | **Typical intensity / relationship** |
| --- | --- | --- | --- | --- | --- | --- |
| Metabolic / endocrine | Hypoglycaemia (<3.0 mmol/L) | 5 (50%) | 18 | 5 (62%) | 18 | Mild; possible relationship |
| Gastrointestinal | Nausea, diarrhea, flatulence, lack of appetite, dyspepsia, stomach disease | 7 (70%) | 12 | 5 (62%) | 10 | Mostly mild; probable/possible relationship |
| Infectious / Respiratory / ENT | Common cold, tonsillitis, urinary infection, virus infection, tooth infection, wound in mouth | 5 (50%) | 17 | 6 (75%) | 26 | Mild; generally not related |
| Neurological | Dizziness, fatigue | 1 (10%) | 3 | 1 (12%) | 1 | Mild; generally not related |
| Respiratory / allergy-like | Dyspnea | 1 (10%) | 1 | 0 | 0 | Mild; generally not related |
| Musculoskeletal / technical | Pain in elbow/knees, bruising, injection-related events | 0 | 0 | 3 (38%) | 3 | Mild; generally not related |
| Other / unclassified | Miscellaneous isolated events | 1 (10%) | 1 | 0 | 0 | Mild; generally not related |

*Data are number of subjects with ≥1 event in each category (n, %) and total number of events. Subjects may contribute to more than one category.
Liraglutide group n=10; placebo group n=8.*

**Supplementary figure S1.** **HbA1c over time**

**Figure S1.** HbA1c (mmol/mol) from baseline to week 65 in participants randomized to liraglutide (solid line, closed circles) or placebo (dashed line, open circles). Values represent mean ± 95% CI based on all available measurements at each visit (weeks 0, 2, 4, 6, 10, 22, 34, 46, 52, 58 and 65). Liraglutide was associated with lower mean HbA1c across the study period, whereas trajectories were broadly parallel between groups. Inferential analyses were performed using a linear mixed-effects model with random intercepts and a week×group interaction term (Table 2); P values are exploratory and not adjusted for multiplicity.

**Supplementary figure S2.** **Body weight over time**

**Figure S2:** Body weight (kg) at weeks 0, 6, 52 and 65 in participants receiving liraglutide (solid line, closed circles) or placebo (dashed line, open circles). Values are mean ± 95% CI for all available observations at each time point. Liraglutide was associated with weight loss during the treatment period with partial regain after discontinuation, whereas weight remained relatively stable with placebo. The adjusted between-group difference in change from baseline to week 52 was −5.9 kg (95% CI −9.8 to −2.0; P = 0.0066) in an ANCOVA model (Table 2); P values are exploratory and not adjusted for multiplicity.
